# Prospects for Development of Induced Pluripotent Stem Cell-Derived CAR-Targeted Immunotherapies

**DOI:** 10.1007/s00005-021-00640-7

**Published:** 2021-12-12

**Authors:** Roberta Mazza, John Maher

**Affiliations:** 1 Leucid Bio Ltd, Guy’s Hospital, Great Maze Pond, London, SE1 9RT UK; 2 King’s College London, School of Cancer and Pharmaceutical Sciences, Guy’s Cancer Centre, Great Maze Pond, London, SE1 9RT UK; 3 Department of Immunology, Eastbourne Hospital, Kings Drive, Eastbourne, BN21 2UD East Sussex UK

**Keywords:** Chimeric antigen receptor (CAR), Induced pluripotent stem (iPS) cell, T-cell, NK cell, Macrophage

## Abstract

Technologies required to generate induced pluripotent stem cells (iPSC) were first described 15 years ago, providing a strong impetus to the field of regenerative medicine. In parallel, immunotherapy has finally emerged as a clinically meaningful modality of cancer therapy. In particular, impressive efficacy has been achieved in patients with selected haematological malignancies using ex vivo expanded autologous T cells engineered to express chimeric antigen receptors (CARs). While solid tumours account for over 90% of human cancer, they currently are largely refractory to this therapeutic approach. Nonetheless, given the considerable innovation taking place worldwide in the CAR field, it is likely that effective solutions for common solid tumours will emerge in the near future. Such a development will create significant new challenges in the scalable delivery of these complex, costly and individualised therapies. CAR-engineered immune cell products that originate from iPSCs offer the potential to generate unlimited numbers of homogeneous, standardised cell products in which multiple defined gene modification events have been introduced to ensure safety, potency and reproducibility. Here, we review some of the emerging strategies in use to engineer CAR-expressing iPSC-derived drug products.

## Introduction

**Fig. 1:**
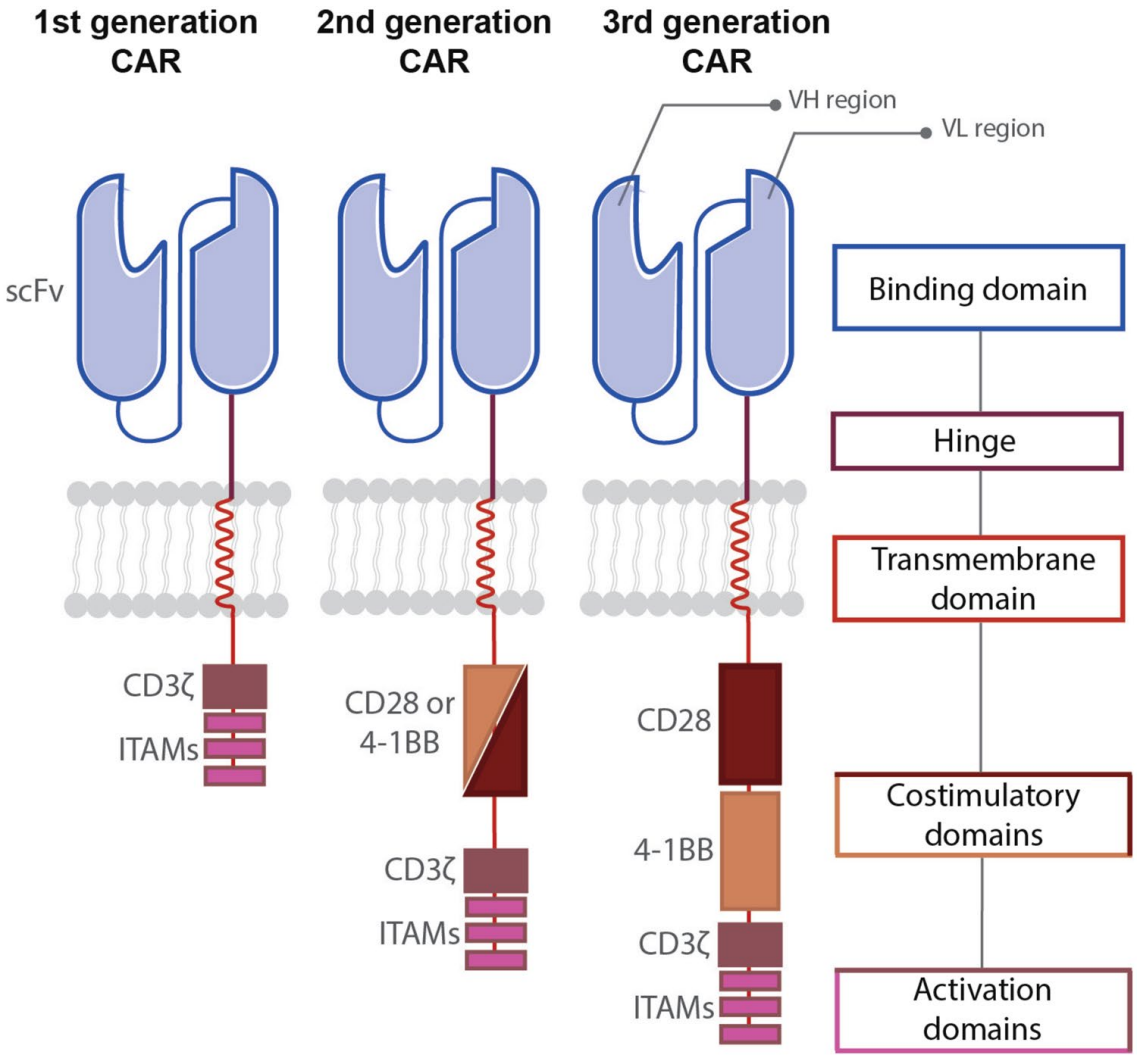
Generations of chimeric antigen receptors. First generation CARs are composed of an antigen-binding domain, a hinge, a transmembrane domain and an intracellular activation domain. Most commonly, the antigen-binding domain consists of an antibody-derived single-chain variable fragment (scFv—shown in blue). The intracellular activation domain usually contains the CD3ζ chain (brown) which contains three immunoreceptor tyrosine activation motives (ITAMs, fuchsia). Second generation CARs contain one additional co-stimulatory domain, most commonly derived from either of CD28 (dark brown) or 4-1BB (beige), while third generation CARs combine two distinct co-stimulatory domains

Despite several successes, many cancers confer poor prognosis and impose a high burden for healthcare systems across the globe (Bray et al. 2020). Nonetheless, optimism has been ignited by the advent of clinically effective immunotherapies for some refractory malignancies over the past two decades (Finck et al. 2020). Despite significant challenges linked to complexity and scalability of manufacture, lack of availability at the time of patient need, high cost and substantial toxicity, cell-based therapy has become the largest area of drug development within the immuno-oncology sector (Xin et al. 2019). This transition has been energised by the expanding toolbox of genetic modification approaches that are now available, leading to new opportunities for precision cancer medicine. One prominent strategy entails the engineering of immune cells to express a chimeric antigen receptor (CAR). These synthetic fusion receptors enable immune cells such as T cells, natural killer (NK) cells or macrophages to bind specifically to a native cancer cell surface marker, triggering multiple cellular effector activities. Prototypic CAR designs were described more than 30 years ago and first entailed the substitution of variable domains of an antigen-specific T-cell receptor (TCR) with the corresponding domains derived from the heavy and light chains of a monoclonal antibody (Kuwana et al. 1987). Eshhar et al. (1993) simplified this design through the introduction of a single chain antibody fragment (scFv) as a targeting moiety to confer antigen specificity. In addition to a targeting moiety, CARs contain a hinge/spacer domain, a transmembrane domain and a bespoke intracellular signalling domain (Fig. 1). The most clinically effective designs are so-called second generation constructs in which the CAR endodomain contains a fused activating and co-stimulatory domain. Activation is generally delivered by CD3ζ, while co-stimulation is most commonly provided by either CD28 (Finney et al.^1998^) or 4-1BB (Finney et al. 2004; Imai et al. 2004) (Fig. 1).

Immunotherapy using patient-derived CAR T cells has achieved major impact in the treatment of selected haematological cancers. To date, five CAR T-cell products have been approved by the United States Food and Drug Administration for the treatment of relapsed refractory B-cell malignancy or multiple myeloma. These comprise the CD19-specific products Tisagenlecleucel (Tisa-cel; Kymriah^®^, Novartis), Axicabtagene ciloleucel (Axi-cel; Yescarta^®^; Kite, Gilead), Brexucabtagene autoleucel (Brex-cel; Tecartus^®^, Kite Pharma/Gilead), Lisocabtagene maraleucel (Liso-cel; Breyanzi^®^, Juno Therapeutics) and a B-cell maturation antigen (BCMA)-specific product called Idecabtagene vicleucel (Ide-cel; Abecma^®^, Bristol Myers Squibb). Many of these drugs have been approved by other international regulatory agencies including the European Medicines Agency, the United Kingdom Medicines and Healthcare products Regulatory Agency, the Canadian Food Inspection Agency and the Australian Therapeutic Goods Administration. Moreover, a sixth CD19-directed product designated ARI-001 has recently been approved by the Spanish Agency of Medicines and Medical Devices (https://www.clinicbarcelona.org/en/news/aemps-authorises-hospital-clinics-car-t-ari-0001-for-patients-with-acute-lymphoblastic-leukaemia, accessed 11 May 2021).

All CAR T-cell drugs currently marketed are patient-derived autologous products, meaning that a single batch of drug treats only one patient. Such an approach leads to high cost, poor scalability, inadequate standardisation, heightened risk of sub-optimal product quality and delay in patient treatment imposed by the time required for product manufacture (Fiorenza et al. 2020; Lyman et al. 2020). Emphasising this, a number of US studies have assessed the cost-effectiveness of Tisa-cel for B-acute lymphoblastic leukaemia (B-ALL) (Lin et al. 2018; Sarkar et al. 2019; Whittington et al. 2018) and both Tisa-cel (Lin et al. 2019) and Axi-cel for large-B cell lymphoma (Roth et al. 2018; Whittington et al. 2019). All raised serious concerns over the value and affordability of CAR T-cell immunotherapy.

A further major obstacle in the clinical application of CAR-based immunotherapy is lack of efficacy in patients with solid tumours, a finding that is attributable to multiple factors (Hull and Maher 2021). First, there is a dearth of tumour-selective antigens that can be safely targeted. Second, in contrast to blood cancers, success requires that the cells can locate and infiltrate within solid tumour deposits. Third, the microenvironment established by solid tumours is highly immunosuppressive owing to nutrient deprivation, hypoxia and infiltration of a range of immunosuppressive leukocytes and mesenchymal stromal cells. A summary of clinical trial activity in the solid tumour arena has recently been published (Adami and Maher 2021). Increasing efforts are being applied to address relevant obstacles in this quest, including target heterogeneity and lack of cancer selectivity, inadequate homing to and penetration of CAR-engineered cells within malignant lesions and the profoundly immunosuppressive nature of the tumour microenvironment (Glover et al. 2021; Hull and Maher 2021). These efforts are likely to make an impact in the near future, as evidenced by recent successes in selected solid tumour types (Glover et al. 2021; Hull and Maher 2021). Given that over 90% of human cancers are solid tumours, the development of an effective CAR-based solution that can impact in this arena will prove profoundly disruptive, but will also impose an substantial drug delivery challenge which autologous solutions are unlikely to overcome.

One strategy that may help to address this forthcoming bottleneck entails the development of universally applicable CAR therapies that can be manufactured at scale. When compared to bespoke patient-derived products, “off-the-peg” CAR-engineered effector cells afford the opportunity to treat multiple patients using a single batch of drug. A further advantage is the fact that dosing units would be available for patients at the time of need.

Allogeneic therapies generated from healthy donor-derived immune cells have attracted great interest in this regard. The advent of genome editing technologies means that it is now possible to disable many barriers to safe infusion of allogeneic T cells, such as elimination of αβ TCR expression, thereby obviating risk of graft versus host disease (GvHD). Although at an early stage of development, recent clinical data has confirmed the promise of this approach in patients with B-ALL (Benjamin et al. 2020). Nonetheless, only small numbers of contaminating cells that retain αβ TCR expression can mediate GvHD (Qasim et al. 2017). Alternatively, cells that lack an αβ TCR such as NK cells, γδ T cells or invariant NKT cells may be used as universal CAR hosts (Halim et al. 2017; Klichinsky et al. 2020; Liu et al. 2020; Rotolo et al. 2018). Moreover, virus-specific T cells offer an alternative off the shelf solution with significant capacity for scalable manufacture and minimal GvHD risk (Aftab et al. 2020).

## Induced Pluripotent Stem Cell Technology

While allogeneic cell products are already demonstrating clinical promise, these strategies remain subject to donor variability, leading to batch to batch heterogeneity. To address this, it is desirable to utilise a single renewable cell source to standardise the manufacture and quality of these novel pharmaceuticals. One approach that has the potential to achieve true consistency and unlimited scalability involves the development of CAR-engineered induced pluripotent stem cells (iPSCs) (Nianias and Themeli 2019). The potential for iPSC-related technology in regenerative medicine is widely appreciated owing to the unique capacity of these cells to undergo unlimited self-renewal and to differentiate into all adult tissue types (Yamanaka and Blau 2010). Almost all somatic cell types have been successfully re-programmed to achieve pluripotency through the introduction of specific sets of reprogramming factors. These comprise the co-expression of OCT3/4, SOX2, KLF4 and c-MYC (Takahashi et al. 2007) or OCT4, SOX2, NANOG, and LIN28, a combination that is believed to be less tumorigenic (Sadeqi Nezhad et al. 2021; Yu et al. 2007). Delivery of reprogramming factors may be achieved using traditional integrating viral vectors (e.g., retrovirus or lentivirus) or non-integrating systems (e.g., episomal, minicircle or transposon-based vectors, Sendai virus, plasmids, mRNA or microRNAs) that minimise risk of insertional mutagenesis (Sadeqi Nezhad et al. 2021). Successful generation of iPSCs can be confirmed by the detection of a panel of cell surface and intracellular markers associated with pluripotency (Sadeqi Nezhad et al. 2021).

**Fig. 2:**
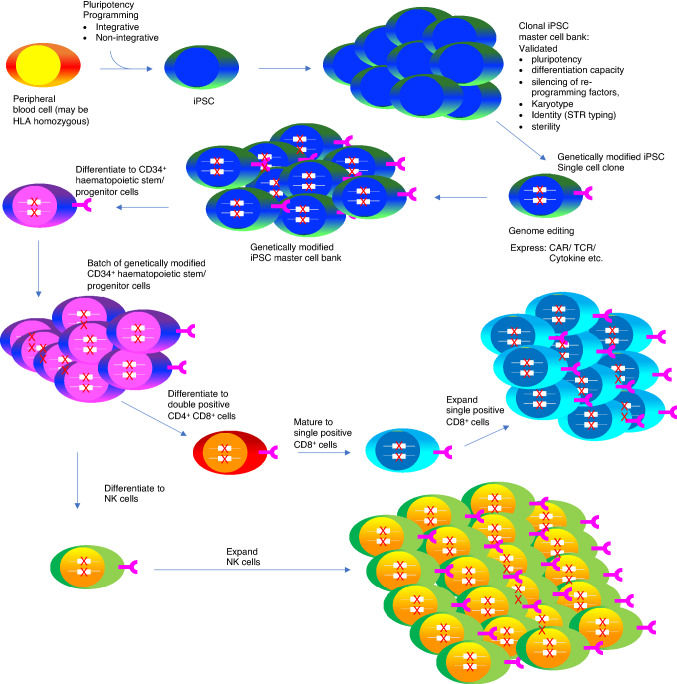
Overview of the production of iPSC-derived CAR T and NK cells. Somatic cells (commonly derived from peripheral blood) are engineered by the introduction of pluripotency-associated genes to generate an iPSC line which is then used to generate a carefully characterized iPSC master cell bank. This can then be subjected to genetic modification enabling the introduction of exogenous genes (e.g., that encoding for a CAR) and/or genome editing (e.g., to reduce immunogenicity of the cells). Subsequently, the gene-edited clone is expanded to develop a master cell bank. These cells may be differentiated to generate CD34^+^ haematopoietic stem cells which in turn can be further differentiated to generate mature functional T cells or NK cells

Given that pluripotent stem cells can be reprogrammed from terminally differentiated hematopoietic cells, nuclear reprogramming of mature lymphocytes into iPSCs has attracted significant interest from the perspective of development of off-the-shelf cellular immunotherapies. This has been accomplished for murine B cells (Hanna et al. 2008), followed quickly thereafter by murine T cells (Watarai et al. 2010) and human T cells (T-iPSCs) (Brown et al. 2010; Loh et al. 2010). Induced pluripotent stem cells derived from hematopoietic cells offer a number of attractive translational opportunities. First, they retain an epigenetic memory of their tissue of origin that can be used to influence differentiation propensity (Kim et al. 2010). Consequently, they are more likely to differentiate to a specific tissue fate such as myeloid and lymphoid lineages when cultured in an appropriate cytokine cocktail (Kim et al. 2010). Second, iPSCs are easier to source than embryonic stem cells and are potentially available from a wider range of donors. This may facilitate better immunological matching of products with patients, for example through improved human leukocyte antigen (HLA) recipient matching and killer immunoglobulin-like receptor (KIR) (mis)matching (Staerk et al. 2010) where required. Third, iPSCs are amenable to multiple genetic modification events which may be used to confer desirable properties upon derived cell products, such as tumour targeting specificity, resistance to immune rejection or susceptibility to elimination via suicide gene systems. Moreover, genetic engineering can be conducted in a single setting, rather than the current requirement for individualised gene modification for each autologous product. This opportunity also eliminates batch to batch variability that may arise due to genetic editing. Fourth, iPSC cell banks are amenable to extensive characterisation to ensure safety and suitability for clinical use (Sadeqi Nezhad et al. 2021) (Fig. 2). Fifth, several advanced feeder-free culture systems that entail the use of chemically defined components have been developed in recent years to facilitate the reproducible propagation and expansion of iPSCs. Such systems are more compatible with automated closed cell culture systems, simplifying production in compliance with current good manufacturing process (cGMP) regulations (Arias et al. 2021; Fan et al. 2014; Paccola Mesquita et al. 2019). Therefore, due to their flexibility of expansion and versatility, iPSC lines derived from peripheral blood cells could provide an unlimited source of pluripotent cells capable of differentiating into functional tumour-specific T cells, NK cells and macrophages in vitro (Huang et al. 2019).

## iPSC-Derived CAR T Cells

In seeking to integrate CAR and iPSC technologies, it was logical to first attempt to generate iPSC-derived CAR T cells. Despite considerable efforts, in vitro generation of T cells from many human pluripotent stem cells proved challenging, prompting efforts to re-program T cells themselves to become iPSCs (T-iPSCs) (Montel-Hagen and Crooks 2019). Systems may involve the production of CD34^+^ haematopoietic stem cells from so-called embryoid bodies, spontaneous aggregates which form when iPSCs are cultured in suspension without feeder cells. Alternatively, CD34^+^ differentiation may be prompted by culture of iPSCs on stromal cells (e.g., OP9 or lines derived from the aorta-gonad-mesonephros region) in the presence of morphogens that favour mesoderm specification followed by hemogenic endothelial specification. The resultant CD34^+^ cells are then replated on monolayers that provide supportive factors such as interleukin (IL)-7 and stem cell factor in addition to Notch signalling via Delta-like ligands (DLL) 1 or 4, thereby promoting T-cell differentiation (e.g., OP9-DLL1 or DLL4). Alternatively, artificial thymic organoid cultures (ATOC) may be generated to achieve this step. This involves the culture of iPSC-derived embryonic mesodermal progenitors with stromal cells (e.g., MS5) that have been engineered to express human DLL1 or DLL4. Importantly, iPSC-derived re-differentiated human T cells are highly proliferative naïve cells with increased telomere length and maintained effector function (Nishimura et al. 2013). Nonetheless, in many cases these cells are not truly identical to primary human T cells. For example, they may lack molecules such as CD2, CD5 and CD28, while exhibiting atypical expression of CD56 or γδ T-cell features (Minagawa et al. 2018; Nishimura et al. 2013; Themeli et al. 2013, 2015; Vizcardo et al. 2013). Interestingly, previous studies in transgenic murine models have proposed that T-cell differentiation depends on thymic signals via Notch and TCR and thus a skew development between αβ versus γδ can be explained by differences in Notch signal strength (Garbe et al. 2006; Vizcardo et al. 2019). Recently, methods have been described to generate early memory phenotype iPSC-derived CD8^+^ T cells (Kawai et al. 2021). One described an alternative approach to rejuvenate T cells entails electroporation of effector cells with mRNA encoding for eight selected transcription factors (LEF1, KLF7, ID3, EOMES, BCL6, TCF7, FOXP1, and FOXO1), promoting reverse differentiation of these cells (Lu et al. 2020). Yet the generation of T lymphocytes from iPSC is a multi-step differentiation process which is variably efficient and thus, makes challenging clinical applications (Nishimura et al. 2013; Themeli et al. 2013; Vizcardo et al. 2013).

A further challenge imposed by the generation of iPSC-derived CAR T cells relates to the occurrence of random TCR rearrangements during iPSC to lymphoid differentiation, giving rise to cells with unpredictable potential for alloreactivity. Use of T cells of defined specificity as starting material helps to circumvent this issue since derived T-iPSCs and single positive CD8^+^ T cells retain the endogenous rearranged αβ TCR (Nishimura et al. 2013; Vizcardo et al. 2013). Accordingly, T-cell clones with specificity for a variety of tumour antigens have been converted to T-iPSC, and thereafter differentiated to generate cytotoxic T cells that retained original target reactivity (Maeda et al. 2016; Minagawa et al. 2018; Vizcardo et al. 2013). Alternatively, clinical grade non T-cell derived iPSCs may be used as starting material following transduction to express a TCR of appropriate specificity (Iriguchi et al. 2021). This approach benefits also from the fact that de novo iPSC generation is not required. Nonetheless, there remains a possibility that further TCR re-arrangements could occur during differentiation (Minagawa et al. 2018; Themeli et al. 2015), a risk that has been attenuated through inactivation of a *RAG* recombinase gene in the iPSCs (Minagawa et al. 2018). Alternatively, genome editing approaches such as deletion within the *TCRA* gene (encodes TCRα) or insertion of a CAR gene at that locus also offer the potential to circumvent risk of toxicity mediated by the endogenous TCR (Eyquem et al. 2017).

An additional constraint to the development of a bank of clinical grade iPSC lines is the potential immune rejection of these allogeneic products. For this reason, selection of HLA homozygous donors may be preferred to simplify the process of recipient matching. To determine the number of donors required to construct a bank of HLA-haplotype-matched iPSCs in discrete populations, probabilistic models have been developed (Gourraud et al. 2012). In consideration of the highly polymorphic nature of the HLA system, Nakatsuji et al. (2008)estimated that an iPSC bank with 50 HLA-homozygous iPSC lines would provide a match for approximately 73% of the Japanese population. Similarly, Taylor et al. (2012) suggested that 93% of the UK population would find a fully matched donor in a bank containing 150 HLA-homozygous lines. However, requirements for comprehensive population matching in some ancestries such as African Americans are likely to be considerably higher. As an alternative to the use of HLA homozygous iPSC, genome-editing technologies may be used to simplify requirements for donor histocompatibility (Xu et al. 2019). Using a strategy that combined biallelic depletion of HLA-A and HLA-B with retention of a single HLA-C haplotype, they estimated that most of the world’s population would find a match within a bank of 12 iPSC master cell lines. HLA-C allotypes fall into two groups (C1 and C2), both of which serve as important ligands for inhibitory and activating KIRs found on NK cells. Moreover, to further reduce risk of immune rejection, this approach could be linked with HLA-class II depletion by inactivation of the class II major histocompatibility complex transactivator gene, *CIITA* (Xu et al. 2019). However, one difficulty that may arise with this solution is the potential for homozygous C1 or C2 cells to be recognised and killed by NK cells that are heterozygous for C1/ C2 (Ichise et al. 2017). In an alternative approach, “invisible iPSC-derived T cells” were recently generated through deletion of β_2_-microglobulin, *CIITA* and the NK cell–ligand poliovirus receptor CD155, accompanied by expression of the NK cell-inhibitory ligand HLA-E, thereby minimising recognition by recipient cytotoxic T cells and NK cells (Wang et al. 2021).

The first application of iPSC technology for the generation of CAR T cells was described by the group of Michel Sadelain (Themeli et al. 2013). Healthy donor peripheral blood T cells were engineered by retroviral transduction to express KLF4, SOX2, OCT-4 and C-MYC, after which multiple T-iPSC clones were isolated. One clone was engineered to express a CD19-specific CAR by lentiviral transduction and then induced to undergo embryoid body formation followed by T-cell differentiation using Notch-expressing feeder cells and appropriate cytokine combinations. Function of iPSC-derived CAR T cells was demonstrated in vitro and in tumour xenograft models. Using gene expression analysis, they showed similarity of the cells to γδ T cells, although they maintained expression of the endogenous αβ TCR.

Traditional methods used to generate iPSC-derived T cells have employed a range of feeder cell lines, as summarised above. However, this imposes significant obstacles to the design of cGMP compliant manufacturing processes. Recently, a feeder-free method has been described with potential to allow efficient and scalable T-cell generation from iPSCs (Iriguchi et al. 2021). Starting material used in this method was either a T-cell clone or an iPSC that had been engineered to express a defined TCR. Notably, introduction of a TCR into an iPSC of non T-cell origin also enhanced T-cell commitment compared to unmodified iPSC. T-cell differentiation was facilitated by the inclusion of CXCL12 and a p38 MAPK kinase inhibitor in the culture. Functionality of CD19 CAR-expressing cells was demonstrated using this system. However, the culture process required 4 months for completion, a drawback that the authors suggest can be circumvented by the generation of a cell bank which is used later for downstream modification such as CAR engineering. Moreover, anti-tumour activity of these iPSC derived CAR T cells was less convincing than that of primary T cells engineered to express the same CAR. This may reflect the fact that CD4^+^ T cells were not present in the iPSC derived cultures (Iriguchi et al. 2021). Given these findings, there has been interest in the development of technologies that permit the generation of single positive CD4^+^ T cells from iPSCs, for example using ATOC systems (Yano et al. 2019).

Clinical translation of iPSC-derived CAR T cells is being led by a number of companies worldwide. To date, Fate Therapeutics is uniquely capable of generating large quantities of uniformly multi-edited off-the-shelf CAR T-cell products. The company developed its own iPSC-product platform and stage-specific T-cell differentiation protocol without the need for feeder cells or serum. Consequently, it can generate fully characterized clonal iPSC lines that can be expanded and achieve  > 100,000-fold expansion in a highly scalable manufacturing process (Knorr et al. 2013). Currently, Fate Therapeutics has developed FT819 in which a CD19 CAR gene has been inserted into the TCRα (*TRAC*) locus to achieve more regulated CAR expression and abrogating risk of GvHD. The CAR endodomain contains an attenuated CD3ζ sequence in which only the membrane proximal immunoreceptor tyrosine-based activation motif (ITAM) retains functionality, a modification that reduces exhaustion of engineered primary T cells (Feucht et al. 2019). In in vitro functional studies, FT819 showed antigen-dependent mechanisms including cytokine release and cytotoxicity activity, while, in in vivo xenograft models of disseminated lymphoblastic leukaemia, this product demonstrated enhanced tumour burden control and extended survival rate compared to primary CAR19 T cells (Chang et al. 2019). A multicentre phase 1 study (NCT04629729) has been designed for patients with relapsed refractory B-cell lymphomas, chronic lymphocytic leukaemia (CLL) and pre-B ALL (Park et al. 2020). Treatment of the first ALL patient in this study was announced recently (https://ir.fatetherapeutics.com/news-releases/news-release-details/fate-therapeutics-announces-treatment-first-patient-landmark, accessed 13 September 2021). Other companies with advancing plans for clinical translation of related technologies include Takeda/ Kyoto University, Mesoblast/Cartherics and Notch/Allogene.

## iPSC-Derived NK Cells and Engineered Derivatives

NK cells are HLA-unrestricted cytotoxic lymphocytes of the innate immune system (Morvan and Lanier 2016) that express an array of germline-encoded activating and inhibitory receptors (Long et al. 2013). The balance between the signals delivered by these receptors determines whether NK cells undergo activation or remain quiescent. Unlike HLA-restricted T cells, NK cells do not require priming by antigen. However, infused NK cells undergo low levels of expansion and achieve poor persistence in vivo, limitations that have been linked to suboptimal therapeutic efficacy (Miller et al. 2005). To potentiate clinical impact, NK cells have been engineered to express CARs which add antigen specificity to the innate anti-tumour activity of these cells. In support of this approach, promising clinical data have recently been presented when CD19 CAR re-programmed allogeneic (umbilical cord blood-derived) NK cells were administered to patients with B-cell non-Hodgkin’s lymphoma or CLL (Liu et al. 2020). Moreover, while CAR T cells commonly induce significant toxicities such as cytokine release syndrome (CRS), neurotoxicity and, in the allogeneic setting, GvHD, these events have not been seen following the adoptive transfer of NK cells (Handgretinger et al. 2016; Liu et al. 2020). Given the limited numbers of NK cells that can be generated using alternative sources, there has been considerable interest in the use of iPSC technology to enable the large-scale production and banking of these cells (Cichocki et al. 2020; Hermanson and Kaufman 2015; Ueda et al. 2018). While many protocols used in the production of T cells from iPSCs are deemed inefficient, generation of iPSC-derived NK cells (iPSC-NK) is now considered a routine activity, whereby iPSC are initially differentiated to haematopoietic progenitors and thereafter are cultured in IL-3, IL-7, IL-15, stem cell factor and FLT3 ligand to induce NK cell formation (Knorr et al. 2013). Early methods employed stromal cell lines in both of these steps (Ni et al. 2011). Alternatively, somatic cells have recently been reprogrammed using pluripotency transcription factors to generate NK cells (Kim et al. 2021). More recently, systems to expand iPSC-NK cells without feeder cells (Knorr et al. 2013; Matsubara et al. 2019; Ueda et al. 2020) and obviating the need for embryoid body formation and CD34^+^ cell purification (Zeng et al. 2017) have been reported. Expanded iPSC-NK cells express a typical array of NK cell markers including NKp30, NKp46, NKG2D, KIR, NKG2A and DNAM-1 although individual levels vary depending on the state of differentiation of the culture. Moreover, transcriptional similarity of iPSC-NK cells and primary NK cells has been demonstrated in some studies (Cichocki et al. 2020).

Preclinical anti-tumour activity of iPSC-NK cells has been demonstrated by a number of investigators. Equivalent anti-tumour activity of iPSC-NK cells and expanded peripheral blood-derived NK cells was shown in xenograft models of epithelial ovarian cancer (Hermanson et al. 2016). Cichocki et al. (2020) also demonstrated that iPSC-NK cells had anti-tumour activity against diverse hematologic and solid tumor cell lines and controlled tumour growth and extended median survival rates in xenograft models of ovarian cancer. In addition, they found that these cells favour local T-cell infiltration and activation, potentially rendering a “cold” tumour hot. This effect could be further capitalised upon by the addition of PD1 blockade, leading to greater anti-tumour activity (Cichocki et al. 2020).

Clinical translation of iPSC-NK cells has been undertaken by Fate Therapeutics, who have developed a suite of therapeutic candidates that are currently undergoing clinical investigation. FT500 is a first-of-kind off-the-shelf NK cell product derived from a human clonal master iPSC line. In a multi-centre Phase 1 study, FT500 has been administered to patient with advanced solid cancers and lymphomas (ClinicalTrials.gov: NCT03841110) (Hong et al. 2020b). Two treatment regimens have been employed. First, FT500 was administered as monotherapy in patients who are eligible for salvage therapy. Alternatively, in patients who have previously failed or progressed on immune checkpoint inhibitor therapy, FT500 was combined with one of three approved immune checkpoint inhibitors (nivolumab, pembrolizumab, or atezolizumab). In each case, FT500 treatment consisted of three once weekly doses (days 1, 8 and 15), each of which was infused following 2 days of lymphodepletion (fludarabine 25 mg/m^2^ and cyclophosphamide 300 mg/m^2^). For patients who achieved disease stabilisation at day 29, there was an option to administer a second treatment cycle of three once weekly doses without lymphodepletion. In the dose escalation phase of the study, two dose levels were evaluated, namely, 100 and 300 million cells per dose. Fifteen patients were treated, of whom 13 initiated a second dosing cycle. Treatment was well tolerated, without evidence host immune rejection, dose-limiting toxicities or severe adverse reactions such as GvHD, CRS or neurotoxicity (Hong et al. 2020a). However, best response was stable disease, which was seen in 11 patients.

NK cells naturally express CD16a (FcRγIIIa), an activating receptor that binds the Fc portion of IgG antibodies and mediates antibody-dependent cell-mediated cytotoxicity (ADCC). Two CD16a variants have been described, namely, the minority 158 V and majority 158F polymorphism, that, respectively, elicit high or low binding affinity for IgG Fc (Ravetch and Perussia 1989). However, cell surface expression of CD16a on activated NK cells is downregulated by a disintegrin and metalloprotease-17 (Romee et al. 2013). To prevent CD16a down-regulation and to enhance the binding of tumour-targeting antibodies, Zhu et al. (2020b) mutated this cleavage site (S197P) in the high affinity 156 V variant to generate a non-cleavable derivative (hnCD16a). This receptor mutant was engineered into iPSC to create a source of hnCD16a-iPSC-NK cells (Zhu et al. 2020b). Stabilised expression of CD16 improved ADCC in pre-clinical models of haematological malignancies and solid tumours, when used in conjunction with anti-CD20 and anti-HER2 monoclonal antibodies, respectively (Zhu et al. 2020b).

To translate this approach to the clinic, FT516 has been derived from a clonal master iPSC line engineered to express the cleavage resistant hnCD16a 158 V variant. FT516 is under evaluation in a Phase 1 study (clinicaltrials.gov: NCT04023071) as a monotherapy for acute myeloid leukaemia (AML) and in combination with CD20-specific monoclonal antibody therapy in patients with advanced B-cell lymphoma. Preliminary clinical results obtained in lymphoma patients in combination with rituximab have recently been published (Strati et al. 2021). Patients received two cycles of treatment consisting of lymphodepletion (fludarabine 30 mg/m^2^ and cyclophosphamide 500 mg/m^2^, each for 3 days), a single dose of rituximab followed by three weekly cycles of FT516 (dose range 30–900 × 10^6^ cells) accompanied by IL-2 (six million IU after each dose of FT516). Six patients had been treated (two each at 30, 90 and 300 × 10^6^ cells). Treatment was well tolerated, without evidence host immune rejection, FT516-related toxicities of grade 3 or above, or episodes of CRS, neurotoxicity or GvHD. Although all patients had received greater than one prior rituximab containing regimen, three of four patients treated with ≥ 90 × 10^6^ cells achieved an objective response (two complete responses, one partial response). Further studies are planned or underway with additional antibody combinations, including the anti-PD-L1 monoclonal antibody, avelumab (clinicaltrials.gov: NCT04551885).

A related iPSC-NK strategy is in development for the treatment of multiple myeloma and benefits from the homeostatic role of IL-15 in the maintenance of NK cells. In FT538, hnCD16a is co-expressed with an IL-15/IL-15 receptor (IL-15R) α fusion protein (IL-15RF) to enable NK cell activity without the need for exogenous cytokine support (Fujii et al. 2018). Both *IL-15RF* and *hnCD16a* genes were targeted to the CD38 locus to achieve bi-allelic CD38 knockout. By this means, FT538 can be used in combination with the anti-CD38 antibody daratumumab, enabling ADCC of CD38-expressing myeloma cells while avoiding fratricide induced by upregulated CD38 expression in activated NK cells. In pre-clinical testing, FT538 cells demonstrated enhanced in vivo persistence and when combined with daratumumab, this led to increased ADCC and anti-tumour activity compared to antibody treatment alone (Bjordahl et al. 2019). FT538 is currently being tested in an open-label, multi-dose phase 1 clinical trial as a monotherapy for the treatment of AML and in combination with a monoclonal antibody against CD38 (daratumumab) or CD319/ SLAMF7 (elotuzumab) for the treatment of multiple myeloma (clinicaltrials.gov: NCT04614636) (Janakiram et al. 2020).

IL-15 is negatively regulated by cytokine-inducible SH2-containing protein (CIS), encoded by the *CISH* gene (Bottino et al. 2016). In *CISH*^−^/^−^ mice, the resultant upregulation of IL-15 signalling mediated resistance to tumour metastasis in vivo (Delconte et al. 2016). In a second genetic approach to enhance IL-15 signalling, *CISH* has been deleted in iPSC-NK cells. This resulted in significantly increased in vivo persistence and enhanced metabolic fitness of these cells, leading to inhibition of tumour progression in a leukaemia xenograft model (Zhu et al. 2020a).

## iPSC-Derived CAR NK Cells

For many years, it has been known that NK cell specificity can be effectively re-programmed using CARs (Daher et al. 2021). Chimeric antigen receptor-engineered iPSC-NK cells are also in development. The group of Dan Kaufman introduced a CD4/ CD3ζ first generation CAR into iPSC-NK cells and demonstrated that they could suppress replication of the human immunodeficiency virus in CD4^+^ T cells (Ni et al. 2014). HLA homozygous iPSC-NK/innate lymphoid cells have been reprogrammed to recognise glypican 3 (GPC)3) using a third generation CD28/4-1BB/ CD3ζ CAR (Ueda et al. 2020). Anti-tumour activity was confirmed in a GPC3-expressing ovarian cancer xenograft model. Superior disease control was evident when compared to phosphate-buffered saline, although iPSC-NK cells that were unmodified or which expressed an irrelevant CAR were not included. Similarly, iPSC-NK cells have been engineered to express a CAR targeted against epidermal growth factor receptor and demonstrated anti-tumour activity in models of glioblastoma multiforme (Yu et al. 2020).

Intriguingly, emerging evidence indicates that optimal CAR design for iPSC-NK cells may be different to T cells. Illustrating this, Li et al. (2018) engineered iPSC-NK cells to express mesothelin-specific CARs using the PiggyBac transposon system. Notably, they found that in vitro and in vivo anti-tumour activity of iPSC-NK cells that expressed an NK cell-optimised CAR (e.g., NKG2D transmembrane domain followed by a fusion of 2B4 and CD3ζ endodomains) outperformed iPSC-NK cells that expressed a traditional T-cell-optimised third generation CAR (e.g., linear endodomain comprising CD28, 4-1BB and CD3ζ) (Li et al. 2018). Next, they compared iPSC-NK cells that expressed an NK cell-optimised CAR with primary T cells engineered to express the T-cell-optimised third generation CAR. Both approaches achieved broadly comparable anti-tumour activity. However, the iPSC-NK cell approach did not cause significant toxicity in immune compromised mice unlike CAR-engineered T cells, which were linked to severe CRS and “lymphocyte-related pathology”.

Fate Therapeutics are also developing a number of CAR iPSC-NK products. The most advanced of these is FT596 which incorporates three genetically encoded functional attributes. The first of these is a CD19-specific CAR in which the endodomain has been adapted to NK cells (e.g., NKG2D transmembrane domain followed by a fusion of 2B4 and CD3ζ endodomains). This is co-expressed with hnCD16a Fc receptor and IL-15RF, as described above. The CAR enhances anti-tumour activity against CD19 expressing B-cells, while the CD16 receptor increases ADCC (Zhu et al. 2020b), and the IL-15/IL-15r fusion receptor promotes in vivo persistence and expansion of the engineered iPSC-NK cells (Fujii et al. 2018). Mechanistically the dual activation of the CAR19 and hnCD16 targeting receptors, combined with IL-15RF signalling can elicit a deeper and more durable response due to a greater degranulation and cytokine release (Goodridge et al. 2019). In keeping with this, preclinical studies have been shown a higher cytotoxic activity of FT596 when combined in a co-culture assay with rituximab. In addition, FT596 has also been successful to prevent tumour progression and promote survival in xenograft models of B-cell malignancy, accompanied by improved safety when compared to CD19-specific CAR T cells. FT596 is currently being evaluated in a Phase 1 study as a monotherapy and in combination with rituximab for the treatment of advanced B-cell lymphoma, or in combination with obinutuzumab for the treatment of CLL (Clinicaltrials.gov: NCT04245722).

FT536 encompasses four genetically encoded functional attributes. First, the iPSC-NKs express a CAR targeted against the NKG2D ligands, MHC class I chain-related protein A (MICA) and MHC class I chain-related protein B (MICB). MICA/B expression is found on a broad range of solid and hematopoietic tumour cells as a result of cellular stress mechanisms to trigger activation signals in NKG2D-expressing NK and T cells. However, MICA/B are frequently shed as an immune escape mechanism, preventing recognition and destruction of tumour cells by the immune system. To circumvent this, Ferrari de Andrade et al. (2018) developed monoclonal antibodies targeted against the MICA/B α3 domain, the site of proteolytic shedding. They successfully inhibited tumour growth in immunocompetent mouse models and reduced human melanoma metastases in a humanized mouse model (Ferrari de Andrade et al. 2018). In FT536, this targeting specificity has been incorporated into a CAR. This is accompanied by hnCD16a, IL-15RF and CD38 knockout to mediate resistance to fratricide. Anti-tumour activity alone and in combination with cetuximab, trastuzumab or irradiation has recently been demonstrated (Goulding et al. 2021).

FT573 comprises an anti-B7-H3 (CD276) CAR, IL-15RF, hnCD16, and CD38 knockout. B7-H3 is an immune checkpoint molecule from the B7 family. Although it is over expressed in several kinds of solid and hematologic malignancies, the function of B7-H3 has not been fully characterized. Initially, B7-H3 was thought to be a co-stimulatory molecule for T cell activation (Chapoval et al. 2001), while more recently, it was identified as a strong inhibitor of T-cell proliferation (Chen et al. 2013; Vigdorovich et al. 2013). Moreover, beyond an immunological role, recent findings have shown that B7–H3 directly promotes cancer by mediating tumour angiogenesis and metastasis (Bonk et al. 2020; Zhang et al. 2017), which makes it a valuable target for cancer therapy to reduce cell proliferation, progression, and metastasis (Castellanos et al. 2017).

FT576 contains a CAR targeted against BCMA, co-expressed alongside IL-15RF, hnCD16a and biallelic CD38 knockout. Functionality against models of multiple myeloma was demonstrated recently in pre-clinical in vitro and in vivo studies, both as monotherapy and in combination with daratumumab (anti-CD38) elotuzumab (anti-SLAM7) or anti-CD19. (Goodridge et al. 2020).

It should also be noted that a number of other companies are seeking to develop CAR-engineered iPSC-NK cells. Illustrating this, Cytovia Therapeutics plan an investigational new drug application this year to evaluate unmodified iPSC-NK cells in liver cancer and AML. They also have an active CAR programme focussed on EGFR, GPC3 and CD38 (https://www.nature.com/articles/d43747-021-00014-0, accessed 06/07/2021) and have announced a strategic partnership with Cellectis to develop TALEN gene edited iPSC-NK and CAR-NK cells (https://www.cellectis.com/en/press/cytovia-therapeutics-and-cellectis-partner-to-develop-talen-gene-edited-ipsc-derived-natural-killer-cells, accessed 06/07/2021).

## iPSC-Derived CAR Macrophages

Engineering CAR macrophages is a relatively new avenue for CAR research. Macrophages have a natural propensity to traffic into solid tumours although they are frequently polarised at that site to an immunosuppressive M2 phenotype. By contrast, macrophages that exhibit a pro-inflammatory M1 phenotype have potent phagocytic and cytolytic activity, secrete pro-inflammatory factors and can present antigens to T cells (Condeelis and Pollard 2006). To harness this, iPSC-derived macrophages were engineered to express a CAR in which a CD20 scFv was fused to FcγR1, enabling them to ingest and destroy B-cell leukemic cells both in vitro and in vivo (Senju et al. 2011). Similarly, murine macrophages were engineered to express a family of engineered chimeric antigen receptors to redirect their phagocytic function against cancer cells (Morrissey et al. 2018). Activity was demonstrated using three ITAM-containing signalling units (FcRγ, Megf10 and CD3ζ). More recently, this principle was exemplified using human macrophages (CAR-Ms) that had been engineered using a chimeric adenoviral vector to express a CAR targeted against human epidermal growth factor receptor 2 (HER2) (Klichinsky et al. 2020). Importantly, the CAR-Ms generated using this system maintained an M1 (pro-inflammatory) macrophage phenotype (Klichinsky et al. 2020). A single infusion of human CAR-Ms decreased tumour burden and prolonged overall survival in two solid tumour xenograft mouse models. A clinical trial of this approach is ongoing using HER2 re-targeted CAR-Ms for the treatment of solid tumours with recent release of preliminary study data (https://www.prnewswire.com/news-releases/carisma-therapeutics-presents-data-from-phase-i-clinical-trial-of-ct-0508-a-her2-targeted-car-macrophage-301421642.html, accessed 19 November 2021).

Once again, iPSC technology offers opportunities for the upscaled clinical translation of this technology. Gutbier et al. (2020) have developed a method to employ iPSCs for a high-yield and large-scale production of cells resembling tissue-resident macrophages. These iPSC-derived macrophages were capable of phagocytosis, cellular cytotoxicity, secretion of pro-inflammatory factors and were amenable to genetic modification at the macrophage-like progenitor stage (Gutbier et al. 2020). Zhang et al. (2020) successfully engineered iPSC-derived CAR macrophages (CAR-iMacs) with antigen-dependent anti-cancer functions. They demonstrated anti-tumour activity of the CAR-iMacs that expressed either a CD19 or mesothelin-specific fusion receptor, employing two distinct endodomain configurations (Zhang et al. 2020). Enhanced phagocytosis and pro-inflammatory immune activities were also observed when CAR-iMacs were stimulated by tumour antigens. These data demonstrate proof of concept for the therapeutic utility of CAR-iMac, obtained from a clonal and genetically homogenous population.

## Conclusions

Engineered immune cells derived from iPSCs offer great promise in the development of a truly scalable, reproducible, standardised and more cost-effective solution to CAR-mediated immunotherapy of cancer and potentially other disease types. These cells can be subjected to multiple genetic modification events and can be characterised extensively prior to banking, providing an inexhaustible source of therapeutic cells which can be deployed promptly when required. Use of this platform is also consistent with multiple dosing strategies which are likely to be required in the event that persistence of these allogeneic cells is limited. Nonetheless, clinical implementation of these technologies presents several challenges. Safety is a particular concern since contaminating iPSCs can generate teratomas owing to their pluripotency. Manufacture using feeder-based systems imposes complexity although some feeder-free techniques have been described and robust and reproducible methodologies will be required for clinical implementation. There is also a clear need to confirm that the potency of iPSC-derived therapeutic CAR cells at least matches that of autologous products. Nonetheless, iPSC-derived CAR-mediated immunotherapies offer a competitive platform in the quest for off-the-shelf products that can be efficiently delivered to patients.

## Abbreviations

ADCC: Antibody-dependent cell-mediated cytotoxicity
ATOC: Artificial thymic organoid cultures
B-ALL: B-acute lymphoblastic leukaemia
BCMA: B-cell maturation antigen
CAR: Chimeric antigen receptor
CAR-Ms: CAR-expressing macrophages
CAR-iMacs: IPSC-derived CAR macrophages
CLL: Chronic lymphocytic leukaemia
cGMP: Current good manufacturing process
CRS: Cytokine release syndrome
DLL: Delta-like ligands
GvHD: Graft versus host disease
HLA: Human leukocyte antigen
hnCD16a: Human non-cleavable CD116a
IL: Interleukin
IL-15RF: IL-15 receptor α fusion protein
Ipsc: Induced pluripotent stem cells
iPSC-NK: IPSC-derived NK cells
ITAM: Immunoreceptor tyrosine-based activation motif
KIR: Killer immunoglobulin-like receptor
MICA/B: MHC class I chain-related protein A/B
NK: Natural killer
scFv: Single chain antibody fragment
TCR: T-cell receptor
T-iPSC: T-cell-derived iPSCs
